# Single-cell transcriptional uncertainty landscape of cell differentiation

**DOI:** 10.12688/f1000research.131861.2

**Published:** 2023-07-20

**Authors:** Nan Papili Gao, Olivier Gandrillon, András Páldi, Ulysse Herbach, Rudiyanto Gunawan

**Affiliations:** 1 Institute for Chemical and Bioengineering, ETH Zurich, Zurich, Zurich, 8093, Switzerland; 2 Laboratoire de Biologie et Modélisation de la Cellule, École Normale Supérieure de Lyon, CNRS, Université Claude Bernard Lyon 1, F69364, France; 3 Équipe Dracula, Inria Center Lyon, Villeurbanne, F69100, France; 4 St-Antoine Research Center, Ecole Pratique des Hautes Etudes PSL, Paris, F-75012, France; 5 CNRS, Inria, IECL, Université de Lorraine, Nancy, F-54000, France; 6 Department of Chemical and Biological Engineering, University at Buffalo - SUNY, Buffalo, NY, 14260, USA

**Keywords:** single cell, gene expression, cell differentiation, transcriptional uncertainty, RNA velocity

## Abstract

**Background: **Single-cell studies have demonstrated the presence of significant cell-to-cell heterogeneity in gene expression. Whether such heterogeneity is only a bystander or has a functional role in the cell differentiation process is still hotly debated.

**Methods: **In this study, we quantified and followed single-cell transcriptional uncertainty – a measure of gene transcriptional stochasticity in single cells – in 10 cell differentiation systems of varying cell lineage progressions, from single to multi-branching trajectories, using the stochastic two-state gene transcription model.

**Results:** By visualizing the transcriptional uncertainty as a landscape over a two-dimensional representation of the single-cell gene expression data, we observed universal features in the cell differentiation trajectories that include: (i) a peak in single-cell uncertainty during transition states, and in systems with bifurcating differentiation trajectories, each branching point represents a state of high transcriptional uncertainty; (ii) a positive correlation of transcriptional uncertainty with transcriptional burst size and frequency; (iii) an increase in RNA velocity preceding the increase in the cell transcriptional uncertainty.

**Conclusions: **Our findings suggest a possible universal mechanism during the cell differentiation process, in which stem cells engage stochastic exploratory dynamics of gene expression at the start of the cell differentiation by increasing gene transcriptional bursts, and disengage such dynamics once cells have decided on a particular terminal cell identity. Notably, the peak of single-cell transcriptional uncertainty signifies the decision-making point in the cell differentiation process.

## Introduction

Cell differentiation is the process through which unspecialized stem cells become more specialized. Because of its important roles in development, cellular repair, and organismal homeostasis, the molecular mechanisms of cell differentiation has been the subject of intense scrutiny. Since roughly 50 years ago – along with the promulgation of the central dogma of molecular biology by Francis Crick and the characterization of the lactose operon by François Jacob and Jacques Monod – the existence of a genetic program has become a prevailing explanation for the cell differentiation process. Although the details were originally not defined, at least not formally, such a genetic program purports a constellation of master genes (i.e., transcription factors) that orchestrate the transcription of downstream target genes in a precise spatiotemporal fashion, resulting in long-lasting alterations in the gene expression patterns ( Herskowitz, 1989; Lewis, 1992; Ohno *et al.*, 1979). A notable experimental evidence substantiating this view is the overexpression of myoD inducing a myogenic phenotype in seemingly naive cells ( Davis *et al.*, 1987). Over the past few decades, the repertoire of such master genes across numerous stem cell systems, such as Nanog, Oct4, Sox2, BATF and MyoD, has begun to coalesce ( Papili Gao *et al.*, 2017; Sartorelli & Puri, 2018; Whyte *et al.*, 2013).

Recent advances in single-cell technologies has revealed new aspects of the cell differentiation that are incompatible with the idea of ordered and programmed (i.e., deterministic) gene expression. More specifically, single-cell data paint a stochastic differentiation process that increases cell-to-cell variability of gene expression. Such an observation has been made for a wide variety of cell differentiation systems, including chicken erythroid progenitors ( Richard *et al.*, 2016), erythroid myeloid lymphoid (EML) cells ( Mojtahedi *et al.*, 2016), mouse embryonic stem cells (mESCs) ( Semrau *et al.*, 2017; Stumpf *et al.*, 2017), and human CD34+ cells ( Moussy *et al.*, 2017). Interestingly, a similar increase of gene expression variation was also observed during the de-differentiation of somatic cells into iPSCs ( Buganim *et al.*, 2012). Stochastic gene expression also appears to have a functional role beyond cell differentiation systems. For example, an increase in cell-to-cell variability of gene expression has been reported during a forced adaptation of budding yeast cells to unforeseen challenges ( Braun, 2015).

In 1957, Conrad Waddington proposed the presently well-known epigenetic landscape that likens the cell differentiation process to a ball rolling on a downward sloping surface, starting from a state of high cell potency and ending at one of possibly several states of low cell potency. The landscape itself is shaped by the action of the genes and gene network – depicted in the less-frequently-shown part B of Waddington’s original figure as a network of ropes that are tied to the surface, creating valleys and hills. Although the epigenetic landscape was originally proposed only as a metaphor of how gene regulation governs the cell differentiation process, this landscape has been formalized within the framework of dynamical systems theory ( Huang, 2009; Saez *et al.*, 2022). The valleys in the Waddington’s epigenetic landscape are equated to stable states of a dynamical system, called attractors, while the hills are often interpreted as energetic barriers.

A number of recent studies provided a graphical representation of the differentiation process based on single-cell transcriptomic data that conforms with the Waddington’s epigenetic landscape ( Fard *et al.*, 2016; Guo & Zheng, 2017; Shi *et al.*, 2018; Zhang & Zhou, 2018; Zwiessele & Lawrence, 2017). More specifically, these studies reconstructed the epigenetic landscape from single-cell gene expression data using probabilistic and quasi-potential methods, for example by applying Hopfield neural networks ( Fard *et al.*, 2016; Guo & Zheng, 2017), a cell-density based strategy ( Zhang & Zhou, 2018), network entropy measurements ( Shi *et al.*, 2018) or more recently Large Deviation Theory ( Lv *et al.*, 2014). However, with the exception of Fard *et al.* (2016) and Lv *et al.* (2014), the aforementioned studies produced monotonic descent passages during cell differentiation, mimicking closely the Waddington’s epigenetic landscape metaphor (see for example ( Bhattacharya *et al.*, 2011; Shi *et al.*, 2018)). Also, none of the above studies consider directly the cellular mechanism that generates stochastic gene transcriptional bursts.

In the present work, we aimed to shed light on the gene transcriptional mechanism behind the rise-then-fall trajectory of cell-to-cell variability in gene expression observed during the cellular differentiation process ( Richard *et al.*, 2016). To this end, we analyzed a collection of published single-cell transcriptomic datasets from various cell differentiation systems, comprising both single-cell RT-qPCR (scRT-qPCR) ( Bargaje *et al.*, 2017; Guo *et al.*, 2010; Moignard *et al.*, 2013; Moussy *et al.*, 2017; Richard *et al.*, 2016; Stumpf *et al.*, 2017) and single-cell RNA-sequencing (scRNA-seq) ( Nestorowa *et al.*, 2016; Treutlein *et al.*, 2016). We employed a likelihood-based analysis using a recent method CALISTA (Clustering And Lineage Inference in Single-cell Transcriptomics Analysis) ( Papili Gao *et al.*, 2020). The analysis relied on a mechanistic model of the stochastic gene transcriptional bursts to describe single-cell gene expression distribution. Specifically, we introduced a new concept of transcriptional uncertainty at single cell level, and by applying CALISTA, we reconstructed the transcriptional uncertainty landscapes for the aforementioned cell differentiation systems. Further, by leveraging the stochastic gene transcriptional model behind CALISTA, we were able identify possible mechanisms behind the overt trajectories of cell differentiation on the transcriptional uncertainty landscapes ( Coulon *et al.*, 2010). For two additional single-cell datasets, we also evaluated the single-cell RNA-velocity using the recently published Velocyto method ( La Manno *et al.*, 2018). The two-state model parameter analysis, combined with RNA-velocities, provided insights into the mechanism regulating cell fate decisions, specifically on the role of stochastic gene transcriptions in the differentiation processes and on the possible mechanism generating this stochasticity.

## Methods

### Main steps of CALISTA workflow

Herein, we briefly describe the main steps involved in the calculation of single-cell transcriptional uncertainty using CALISTA ( Papili Gao *et al.*, 2020).

**Pre-processing.** Given an N×G single-cell expression matrix M , where *N* denotes the number of cells and *G* the number of genes, the pre-processing in CALISTA involves two steps: a normalization of the expression data $m_{n,g}$ – i.e. the number of transcripts of gene *g* in the *n*-th cell, and a selection of the most variable genes ( Papili Gao *et al.*, 2020).

**Cell clustering.** CALISTA clustering follows a two-step procedure. The first step involves a greedy optimization strategy to find cell clustering that maximizes the total cell likelihood, i.e. the sum of the likelihood value for all cells. The single-cell likelihood value is computed as the joint probability of the cell’s gene expression data, which is set equal to the product of the probabilities of the mRNA counts for the selected genes based on the mRNA distribution from the two-state stochastic gene transcription model. To avoid issues with numerical overflow, we use the logarithm of the cell likelihood. By performing the greedy optimization multiple times, a consensus matrix containing the number of times two cells in the dataset are put in the same cluster, is generated. In the second and final step, CALISTA generates the cell cluster assignments by using *k*-medoids clustering based on the consensus matrix. The final outcome of CALISTA’s clustering is the assignment of cells into *K* clusters and the optimal model parameters for the two-state gene transcription model: $\theta \left(g,k\right)={\left\{\theta_{\mathrm{on}},\theta_{\mathrm{off}},\theta_{t}\right\}}_{g}^{k}.$ for each gene *g* in cluster *k* ( Papili Gao *et al.*, 2020). In this case, $\theta_{\mathrm{on}}$ is the normalized rate of the promoter activation, $\theta_{\mathrm{off}}$ is the normalized rate of the promoter inactivation, and $\theta_{t}$ is the normalized rate of mRNA production when the promoter is active. These parameters are normalized by the rate constant of mRNA degradation $\theta_{d}$ , so that $\theta_{d}=1$ .

**Lineage progression inference.** In CALISTA, cell lineage progression is inferred based on cluster distances – a measure of dissimilarity between two clusters. The cluster distance of two cell clusters is defined as the average decrease in the cell likelihood value if the cells from these two clusters are grouped as one cluster, as opposed to the original clustering. The lineage progression graph is built by adding transition edges between pairs of clusters in increasing magnitude of cluster distance until all clusters are connected to at least one other cluster, or based on user-specified criteria.

**Single-cell transcriptional uncertainty.** The last step in our analysis is to compute the final single-cell likelihood. Briefly, for each cell, we consider all edges in the lineage progression graph that are adjacent to the cell’s respective cluster, i.e. edges that eminate from or pointing to the cluster to which the cell belongs. The likelihood of a cell along an edge is evaluated by interpolating the likelihood values of the cell’s gene expression using the mRNA distributions from the two adjacent clusters. Each cell is then assigned to the edge along which its interpolated likelihood value is maximum, and the final cell likelihood is set to this maximum value. The single-cell transcriptional uncertainty is evaluated as the negative logarithm of the cell likelihood value (NLL). Following the way single-cell likelihood is computed (see Cell Clustering section above and Papili Gao *et al.*, 2020), the NLL for each cell *n*, denoted by ${\text{NLL}}^{n}$ , is the sum of the NLL from every gene *g* for that cell, i.e. ${\text{NLL}}^{n}=\sum_{g=1}^{N_{g}}{\text{NLL}}_{g}^{n}$ where *N*_*g*_ denotes the number of genes.

**Pseudotimes calculation.** We can evaluate the pseudotimes for the cells according to the following procedure. First, a pseudotime is given to each cluster with a value between 0 (initial cell state) and 1 (final cell fate). Subsequently, we determine the linear fractional position of each cell along its respective edge at which its interpolated likelihood value is maximum (see Single-cell transcriptional uncertainty). The pseudotime of a cell is computed by a linear interpolation of the pseudotimes of the two clusters adjacent to its assigned edge according to the cell’s linear fractional position on this edge.

**Epigenetic landscape reconstruction.** To visualize the 3D transcriptional uncertainty landscape, we apply dimensional reduction techniques such as principal component analysis (PCA) or t-SNE on the z-scored expression data, to project the gene expression of each individual cell on two dimensional axis, which gives the x-y axis of the landscape plot. For the z axis, we plot the NLL values. The transcriptional uncertainty landscape surface is reconstructed by estimating local approximation of individual cell 3D coordinates on a regular 30×30 grid by using a publicly available Matlab (R2020a) surface fitting package called gridfit.

### Pre-processing and analysis of single-cell expression datasets

**Bargaje *et al.* scRT-qPCR dataset.** The dataset includes the expression profiles of 96 genes from 1896 single cells at eight different time points (day 0, 1, 1.5, 2, 2.5, 3, 4, 5) during the differentiation of human pluripotent stem cells (iPSCs) into either mesodermal (M) or endodermal (En) fate ( Bargaje *et al.*, 2017). By employing CALISTA, we obtained five cell clusters and detected a bifurcation event, which gives rise to the two final cell fates. After lineage inference, we pseudotemporally ordered cells along the inferred differentiation paths (for more details, see ( Papili Gao *et al.*, 2020)).

**Treutlein *et al.* scRNA-sequencing dataset.** The dataset includes the gene expression profiles of 405 cells during reprogramming of mouse embryonic fibroblast (MEF) into a desired induced neural (iN) and an alternative myogenic (M) cell fate ( Treutlein *et al.*, 2016). We pre-processed the data using CALISTA to select the 40 most variable genes (10% of the number of cells) for the transcriptional uncertainty analysis. CALISTA identified four different subpopulations and successfully recovered the bifurcation event (for more details, see ( Papili Gao *et al.*, 2020)).

**Richard *et al.* scRT-qPCR dataset.** The dataset contains the expression profile of 91 genes measured from 389 cells at six distinct time points (0, 8, 24, 33, 48, 72 h) during the differentiation of primary chicken erythrocytic progenitor cells (T2EC) ( Richard *et al.*, 2016). Following the CALISTA pre-processing step, we removed cells in which less than 75% of the genes are expressed. Then, we selected the subset of genes with at least one non-zero expression values. A total of 354 cells and 88 genes were considered in the transcriptional uncertainty analysis. Based on eigengap heuristics ( Papili Gao *et al.*, 2020; von Luxburg, 2007), we grouped cells into six optimal clusters and ordered cells along the inferred linear trajectory (see the *Extended data* S8 Figure ( Gao *et al.*, 2023)).

**Stumpf *et al.* scRT-qPCR dataset.** The dataset comprises the single-cell expression of 97 genes at seven time points (0, 24, 48, 72, 96, 120, 168 h) during neural differentiation of mouse embryonic stem cells (E14 cell line) ( Stumpf *et al.*, 2017). In the data pre-processing, we excluded cells in which less than 70% of genes are expressed. Then, we selected genes with at least one non-zero expression values. A total of 276 cells and 93 genes were considered for for the transcriptional uncertainty analysis. Based on eigengap heuristics ( Papili Gao *et al.*, 2020), we grouped cells into five optimal clusters and ordered cells along the inferred linear trajectory ( *Extended data* S9 Figure ( Gao *et al.*, 2023)).

**Moussy *et al.* scRT-qPCR dataset.** The single-cell expression dataset includes normalized Ct values for 91 genes in 435 cells captured at five distinct time points (0, 24, 48, 72, 96 h) during human cord blood-derived CD34+ differentiation ( Moussy *et al.*, 2017). We employed CALISTA to group cells into seven clusters, reconstruct the developmental trajectory and calculate pseudotimes ( *Extended data,* S10 Figure ( Gao *et al.*, 2023)).

**Guo *et al.* scRT-qPCR dataset.** The dataset comprises the single-cell expression values of 48 genes from 387 individual cells isolated at four distinct developmental cell stages, from 8-cell stage mouse embryos to 64-blastocyst ( Guo *et al.*, 2010). By applying CALISTA, we identified seven different subpopulations along the differentiation process, and the inferred lineage hierarchy pinpointed two bifurcations events at 32- and 64-cell stage ( *Extended data,* S11 Figure ( Gao *et al.*, 2023)). The timing of the lineage bifurcations coincides with two well-known branching points: one at 32-cell stage when totipotent cells differentiate into trophectoderm (TE) and inner cell mass (ICM), and another at 64-cell stage when ICM cells differentiate into primitive endoderm (PE) and epiblast (E).

**Nestorowa *et al.* scRNA-sequencing dataset.** The dataset comprises single-cell gene expression of 1656 cells from mouse hematopoietic stem cell differentiation ( Nestorowa *et al.*, 2016). We pre-processed the data by removing genes with non-zero values in less than 10% of the cells. Then, we selected 433 most variable genes, which is 10% of the number of genes after the previous pre-processing step, for the transcriptional uncertainty analysis ( Papili Gao *et al.*, 2020). We set the optimal number of clusters based on the original study ( Nestorowa *et al.,* 2016), which reported six different subpopulations and two bifurcation events: the first one producing common myeloid progenitor (CMP) from lymphoid-primed multipotent progenitors (LMPP), and the second one generating granulocyte–monocyte progenitors (GMP) from megakaryocyte-erythroid progenitors (MEP) ( *Extended data,* S12 Figure ( Gao *et al.*, 2023)).

**Moignard *et al.* scRT-qPCR dataset.** The dataset contains the single-cell expression level of 18 transcription factors measured in a total of 597 mouse bone marrow cells during hematopoietic differentiation. By applying CALISTA, we successfully identified the five subpopulations and the two branching points detected in the original study ( Moignard *et al.*, 2013): long-term hematopoietic stem cells (HSC) differentiating into megakaryocyte–erythroid progenitors (PreM) or lymphoid-primed multipotent progenitors (LMPP); LMPP cells differentiating into granulocyte–monocyte progenitors (GMP) and common lymphoid progenitors (CLP) (for details see ( Papili Gao *et al.*, 2020)).

### Pairwise correlation analysis of transcriptional uncertainty and transcriptional burst size and frequency

We define gene transcriptional burst size and burst frequency using the two-state model parameters, as follows:

$$\text{Burst size S}=\frac{\theta_{t}}{\theta_{\mathrm{off}}}$$ (1)

$$\text{Burst frequency F}=\theta_{\mathrm{on}}$$ (2)

The burst size and burst frequency for each cluster *k* and gene *g*, denoted by *S*_*g*, *k*_ and *F*_*g*, *k*_, respectively, are evaluated using the cluster parameters $\theta \left(g,k\right)={\left\{\theta_{\mathrm{on}},\theta_{\mathrm{off}},\theta_{t}\right\}}_{g}^{k}$ obtained from CALISTA single-cell clustering analysis. Meanwhile, the average gene-wise NLL values for each single-cell cluster is computed as follows:

$${\bar{\text{NLL}}}_{g,k}=\frac{\sum_{n=1}^{N_{k}}{\text{NLL}}_{g,k}^{n}}{N_{k}}\;$$ (3)

where ${\text{NLL}}_{g,k}^{n}$ is the negative log-likelihood of cell *n* based only on the expression of gene *g* in cluster *k*, and *N _k_* is the total number of cells in cluster *k*. Finally, the Pearson correlation coefficients between the burst size *S*_*g*, *k*_ and ${\bar{\text{NLL}}}_{g,k}$ and between the burst frequency *F*_*g*, *k*_ and ${\bar{\text{NLL}}}_{g,k}$ are computed for each dataset to quantify the pairwise associations between these variables. The statistical significance of the correlation coefficients is determined using the t-test—specifically, by evaluating the score $t=r\sqrt{\left(n-2)/(1-r^{2}\right)}$ where *r* is the sample correlation coefficient.

### RNA velocity analysis

Cells and genes were first filtered based on the pre-processing strategy in the original publication by La Manno and colleagues ( La Manno *et al.*, 2018), which resulted in a total of 1720 cells and 1448 genes from human glutamatergic neurogenesis, and a total of 18140 cells and 2141 genes from the mouse hippocampus dataset. We further reduced the number of genes to only the top 500 highly variable genes for the transcriptional uncertainty analysis. The cell cluster assignments generated by Velocyto – the algorithm for computing RNA velocity from the original publication ( La Manno *et al.*, 2018) – were considered, instead of using CALISTA. Based on the clustering, we employed CALISTA to generate the lineage progression and cell pseudotimes ( *Extended data,* S13 Figure ( Gao *et al.*, 2023)). The RNA velocity and transcriptional uncertainty values for the top 500 genes were calculated by employing Velocyto and CALISTA, respectively. The cell-wise RNA velocity was set to the Euclidean norm of the vector of RNA velocities for each cell, while the cell-wise NLLs was

$${\text{NLL}}_{n}^{k}=\frac{\sum_{g=1}^{500}{\text{NLL}}_{g}^{n}}{500}\;$$ (4)

## Results

### Single-cell transcriptional uncertainty landscape

**Figure 1.**
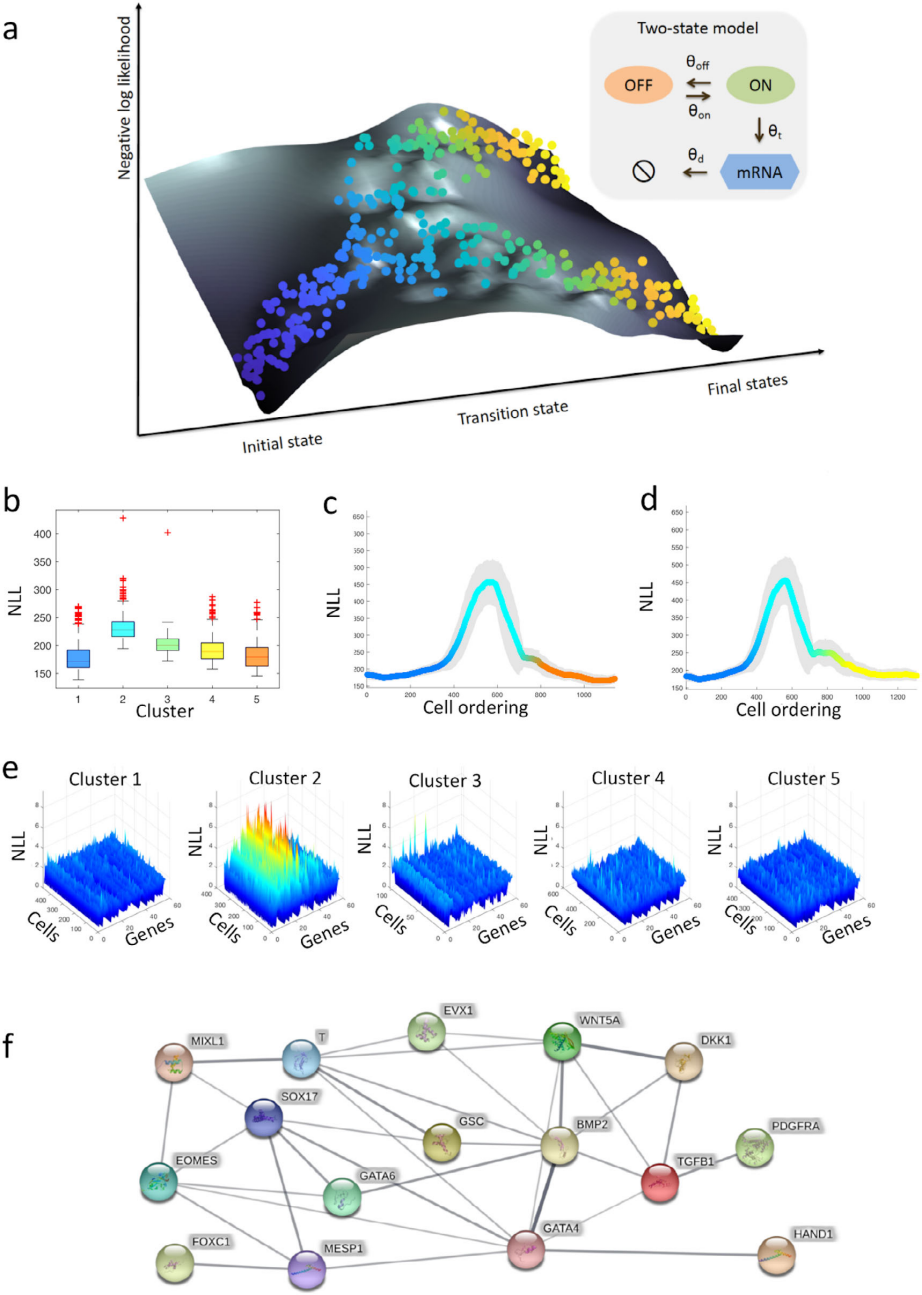
Single-cell transcriptional uncertainty landscape. (a) The illustration depicts the landscape of single-cell transcriptional uncertainty during a differentiation process over the (pseudo) time (from blue to yellow). Each dot corresponds to a cell in the single-cell transcriptomic dataset. Cells start their journey from a valley in the landscape, through a hill, before ending at one of the final valleys/states. (b–f) Analysis of single-cell transcriptional profiles during iPSC differentiation into cardiomyocytes ( Bargaje *et al.*, 2017). (b) Boxplots of the negative log-likelihood (NLL) values for each single-cell cluster. (c–d) Moving-window average NLL along (c) endoderm and (d) mesoderm fate trajectory. (e) NLL of each gene and cell for every single-cell cluster. (f) Protein-protein interaction network of top variable genes inferred by STRING ( Szklarczyk *et al.*, 2015). Blue nodes represent transcription factors, while red nodes denote proteins involved in signal transduction. The width of the edges denotes the confidence for the inferred relationship (thicker edge = higher confidence).

In this work, we used CALISTA ( Papili Gao *et al.*, 2020), a likelihood-based bioinformatics toolbox designed for an end-to-end analysis of single-cell gene expression data, to evaluate the transcriptional uncertainty of each individual cell based on its gene expression data (see the *Extended data*, Supplementary Notes S1 ( Gao *et al.*, 2023)). CALISTA uses the two-state model of stochastic gene transcription bursts to characterize the steady state distribution of mRNA counts in individual cells ( Peccoud & Ycart, 1995). In the model, a gene promoter stochastically switches between the ON and OFF state, and only in the ON state can gene transcription occur. The distribution of mRNA depends on four model parameters: *θ _on_* (the rate of promoter activation), *θ _off_* (the rate of promoter inactivation), *θ _t_* (the rate of mRNA production when the promoter is in the ON state), and *θ _d_* (the rate constant of mRNA degradation) ( Herbach *et al.*, 2017; Kim & Marioni, 2013) (see Figure 1a). For example, when *θ _off_* >> *θ _on_* and *θ _off_* >> *θ _d_*, keeping *θ _t_*/ *θ _off_* fixed, mRNA are produced through bursts of short but intense transcription, which is a typical case observed for gene transcriptions in single cells ( Munsky *et al.*, 2012) ( Suter *et al.*, 2011). As the mRNA distribution is linked to mechanistically interpretable parameters, CALISTA is able to give insights into the possible mechanism driving the cell heterogeneity dynamics during cell differentiation.

CALISTA employs a maximum likelihood approach and assigns a likelihood value to each cell based on its gene expression based on the mRNA distribution governed by the two-state model of stochastic gene transcription. The single-cell transcriptional uncertainty is evaluated as the negative logarithm of the likelihood (NLL) value for a cell. The single-cell likelihood value reflects the joint probability of its gene expression repertoire. A cell with a low likelihood value may indicate that the gene expression of the cell is different from its neighboring cells, i.e. the cell is an outlier. But, more interestingly, a low likelihood value may also correspond to a cell state of high uncertainty in the gene expression. The group of cells in such a high uncertainty state have gene expressions that are dissimilar to each other, and thus, the gene expression distribution will have a high entropy. By visualizing the single-cell transcriptional uncertainty over the two-dimensional projection of the single-cell transcriptomics data—for example, using the first two principal components from PCA—we constructed a transcriptional uncertainty landscape in the form of a surface plot of the NLL value. In this way, we studied the landscape of transcriptional uncertainty during cell differentiation at single-cell resolution. On such single-cell transcriptional uncertainty surface, an aberrant cell can be easily distinguished from a cell of high uncertainty state, since such an aberrant cell will appear isolated from its neighboring cells with a high NLL.

### Transcriptional uncertainty landscape of iPSC cell differentiation to cardiomyocytes

In the following, we demonstrated an application of our procedure described above to a single-cell transcriptional dataset from cardiomyocytes differentiation from human induced pluripotent stem cells (iPSCs) ( Bargaje *et al.*, 2017). The single-cell clustering of CALISTA returned five clusters ( Papili Gao *et al.*, 2020) and identified one bifurcation event in the lineage progression, which led to two cell lineages ( Bargaje *et al.*, 2017), in good agreement with the number of cell types reported in the original study. The estimated uncertainty landscape shows cells exiting the initial epiblast state that is characterized by a valley in the landscape, passing through a hill of high transcriptional uncertainty corresponding to primitive streak (PS)-like progenitor state, before ending up at one of the low transcriptional uncertainty terminal states corresponding to either mesodermal (desired) or endodermal (undesired) fate (see the *Extended data*, S1 Figure ( Gao *et al.*, 2023)). As depicted in Figure 1b, the intermediate cell cluster (cluster 2) comprising PS-like cells have higher cell uncertainty (lower single-cell likelihood) than the other clusters. Figure 1c and d give the moving-averaged uncertainty values for pseudotemporally ordered cells using a moving window of 10% of the total cells for both endodermal and mesodermal paths, respectively. The moving-averaged transcriptional uncertainty for the two differentiation paths follows a rise-then-fall trajectory where the peak of uncertainty coincides with the lineage bifurcation event.

We explored whether the rise-then-fall in uncertainty is an artefact from using the two-state model to evaluate the cell likelihood values. To this end, we implemented a modified version of the algorithm for ordering cells by calculating the cell likelihood values using the empirical (observed) distribution, instead of the analytical distribution from the two-state model. As shown in the *Extended data* S2 Figure ( Gao *et al.*, 2023), the transcriptional uncertainty landscape from the modified implementation shows a strong resemblance to the original one. We also investigated whether the number of clusters may affect the landscape, in which using too few of the clusters may artificially inflate the uncertainty due to the mixing of cells from different states. We reran CALISTA by using a higher number of clusters (set to nine based on the eigengap heuristic ( von Luxburg, 2007)). The hill in the uncertainty landscape is again seen around the bifurcation event upon using a higher number of cell clusters ( *Extended data,* S3 Figure ( Gao *et al.*, 2023)). Finally, we used a different algorithm to cluster cells, specifically using a Laplacian-based clustering algorithm called single-cell interpretation via multikernel learning (SIMLR) ( Wang *et al.*, 2017), to test whether the shape of the transcriptional uncertainty landscape changes with the clustering algorithm. The single-cell clusters can be interpreted as the transitional states that the differentiating cells go through. Starting with the result of SIMLR cell clustering, we then generated the lineage progression and estimated the cell likelihood values using CALISTA. The transcriptional uncertainty landscape from SIMLR cell clustering has the same shape as that in *Extended data* S1 Figure ( Gao *et al.*, 2023), demonstrating that the transcriptional uncertainty landscape observed above is not dependent on using CALISTA for cell clustering ( *Extended data,* S4 Figure ( Gao *et al.*, 2023)).

To further elucidate the role of specific genes in shaping the transcriptional uncertainty landscape, we looked at the transcriptional uncertainty associated with individual genes. Figure 1e depicts the NLL distribution of each gene for the five single-cell clusters. As expected, cells in cluster 2 have generally higher NLL than those in the other clusters. Figure 1e clearly illustrates that within cluster 2, some genes show higher NLL values than the others ( *Extended data,* S5 Figure ( Gao *et al.*, 2023)). To identify the important genes related to transcriptional uncertainty, we identified genes with NLL values exceeding a threshold δ for at least 30% of the cells in each cluster, where δ is set to 3 standard deviation above the overall mean NLL for all cells and genes in the dataset (see Methods equation (2)). None of the genes in clusters 1, 4 and 5 have a NLL above the threshold. Meanwhile, 16 and eight genes in clusters 2 and 3, respectively, pass the above criterion for high uncertainty with four common genes between the two gene sets ( *Extended data,* S1 Table ( Gao *et al.*, 2023)). Genes with high transcriptional uncertainty in cluster 2 may have functional roles in cell fate determination. The gene set of cluster 2 includes known genes upregulated only in the PS-like state (e.g. EOMES, GSC, MESP1 and MIXL1), as well as markers of mesodermal and endodermal cells (e.g. BMP4, HAND1, and SOX17) ( Bargaje *et al.*, 2017; Papili Gao *et al.*, 2020) ( *Extended data,* S6 Figure ( Gao *et al.*, 2023)). Meanwhile, the main contributors to cell uncertainty in cluster 3 (e.g. BMP4 and MYL4 ( Bargaje *et al.*, 2017; Papili Gao *et al.*, 2020)) are known transition genes between PS-like cells and the final mesoderm fate ( *Extended data,* S7 Figure ( Gao *et al.*, 2023)). Figure 1f depicts the protein-protein interaction (PPI) network related to the gene set of cluster 2 using STRING (minimum required interaction score of 0.4) ( Szklarczyk *et al.*, 2015), indicating that these genes form a strongly interconnected hub of known transcription factors and molecules involved in the signal transduction of embryonic development ( *Extended data,* Table S1 ( Gao *et al.*, 2023)).

### Transcriptional uncertainty landscapes of cell differentiation

**Figure 2.**
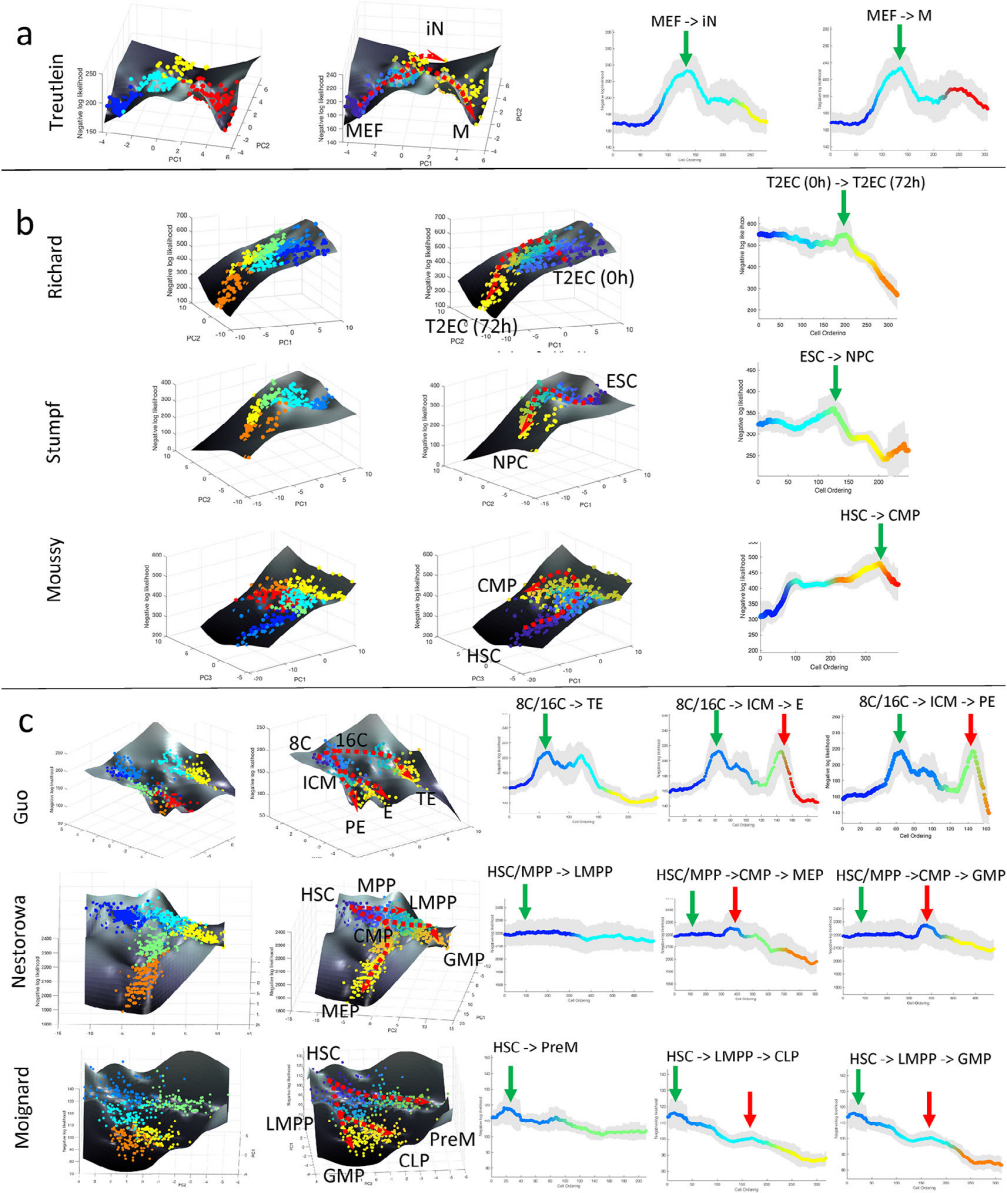
CALISTA analysis of single-cell expression data. (a–c) Landscape plots (based on cell clusters and pseudotime) and moving-averaged negative log-likelihood (NLL) values for each differentiation path of (a) single-branching trajectory ( Treutlein *et al.*, 2016), (b) linear trajectories ( Moussy *et al.*, 2017; Richard *et al.*, 2016; Stumpf *et al.*, 2017), (c) multi-branching trajectories ( Guo *et al.*, 2010; Moignard *et al.*, 2013; Nestorowa *et al.*, 2016). Green and red vertical arrows in moving-averaged NLL plots indicate the first and second peak in cell uncertainty, respectively. Abbreviations: (a) MEF: mouse embryonic fibroblast, iN: induced neuronal, M: myocyte, (b) T2EC: chicken erythocytic progenitor cell, ESC: embryonic stem cell, NPC: neuroprogenitor cell, HSC: haematopoietic stem cell, CMP: common lymphoid progenitor, (c) 8C: eighth cell stage, 16C: sixteenth cell stage, ICM: inner cell mass, TE: trophectoderm, PE: primitive endoderm, E: endoderm, MPP: multipotent progenitor, LMPP: lymphoid multipotent progenitor, CMP: common myeloid progenitor, MEP/PreM: megakaryocyte-erythrocyte progenitor, GMP: granulocyte-monocyte progenitor, CLP: common lymphoid progenitor.

We further applied the procedure above to seven additional single-cell transcriptomic datasets that were generated using scRT-qPCR ( Guo *et al.*, 2010; Moignard *et al.*, 2013; Moussy *et al.*, 2017; Richard *et al.*, 2016; Stumpf *et al.*, 2017) and scRNA-sequencing ( Nestorowa *et al.*, 2016; Treutlein *et al.*, 2016), to assess the universality of the rise-then-fall feature of single-cell transcriptional uncertainty landscape during cell differentiation. The first of these datasets came from 405 cells during mouse embryonic fibroblast (MEF) reprogramming into induced neural (iN) and myogenic (M) cells ( Treutlein *et al.*, 2016). Like the iPSC differentiation above, the lineage progression has a single bifurcation point. As depicted in Figure 2a, the single-cell transcriptional uncertainty increases from the initial MEF state and reaches a peak around the bifurcation before decreasing toward two end-point cell fates. The rise-then-fall of transcriptional uncertainty in the MEF reprogramming is in good agreement with what we observed in the iPSCs differentiation above. Higher entropy of gene expression distribution in a cell population has also been reported in the reprogramming of iPSCs ( Buganim *et al.*, 2012).

Next, we analyzed datasets from cell differentiation processes without a lineage bifurcation and with multiple lineage bifurcations. Three scRT-qPCR datasets came from differentiation systems without bifurcation, including the Richard *et al.* study on chicken erythrocytic differentiation of T2EC cells ( Richard *et al.*, 2016), the Stumpf *et al.* study on differentiation of mouse embryonic stem cells (ESC) to neural progenitor cells (NPC) ( Stumpf *et al.*, 2017), and the Moussy *et al.* study during CD34+ cell differentiation ( Moussy *et al.*, 2017). The single-cell clustering and lineage progression by CALISTA produced the expected cell differentiation trajectory (see *Extended data,* S8 to S10 Figures ( Gao *et al.*, 2023)). The single-cell transcriptional uncertainty landscapes of these three differentiation systems, as shown in Figure 2b, exhibit a rise-then-fall profile, creating a hill that the cells traverse through in the differentiation process. A transitory increase in single-cell gene expression uncertainty was reported either directly or indirectly in the original publications. In Richard *et al.* (2016) and Stumpf *et al.* (2017), the authors adopted the Shannon entropy to quantify cell-to-cell variability (uncertainty), while Moussy *et al.* (2017) reported an unstable transition state with ‘hesitant cells’ flipping their morphology between polarized and round shapes before committing to the common myeloid progenitors-like fate. Morphological uncertainty therefore corresponded to a higher transcriptional uncertainty. Note that the Moussy *et al.* study looked at only the initial phase of the (hematopoietic) cell differentiation, and thus, it is likely that the differentiation process had not completed for the cells in the dataset.

The next set of single-cell gene expression data came from differentiation systems with multi-branching lineage, including the Guo *et al.* study during mouse embryo development from zygote to blastocyst ( Guo *et al.*, 2010), Nestorowa *et al.* (2016) and Moignard *et al.* (2013) studies on hematopoietic stem cell differentiation. Figure 2c shows the single-cell transcriptional landscape for each of the datasets. For the Guo *et al.* study, we identified seven cell clusters and identified two bifurcations in the lineage. Here, we observed two hills in the transcriptional uncertainty landscape, each coinciding with a bifurcation event in the lineage progression – one at 32-cell stage (cluster 2 to cluster 3 and 4) and another at 64-cell stage (cluster 4 to cluster 6 and 7) (see the *Extended data,* S11 Figure ( Gao *et al.*, 2023)). For the Nestorowa *et al.* (2016) ( *Extended data,* S12 Figure ( Gao *et al.*, 2023)) and Moignard *et al.* (2013) (see Methods and ( Papili Gao *et al.*, 2020)) datasets, we again observed peaks in the transcriptional uncertainty landscape that colocalize with the bifurcation points in the lineage progression.

The use of the two-state mechanistic gene transcriptional model within CALISTA enabled us to probe into a mechanistic explanation for the observed shape of the transcriptional uncertainty landscape. Table 1 show the pairwise Pearson correlations between the cell-averaged NLL of each cluster with two biologically interpretable model parameters, namely transcriptional burst size (number of transcripts generated in each burst) and burst frequency (occurrence of burst per unit time) ( Nicolas *et al.*, 2017) (see Methods). The Pearson correlations indicate that the single-cell gene expression uncertainty increases with higher burst size and burst frequency ( *p*-value ≤ 0.01). Higher transcriptional burst size and frequency are associated with a lower *θ*_*off*_ – a lower rate of promoter turning off – and a greater *θ*_*on*_ – higher rate of promoter turning on. One possible explanation for such a change in model parameters is a higher chromatin accessibility during the transition period of cell differentiation. This finding is consistent with the view that stem cells increase its gene expression uncertainty or stochasticity by adopting a more open chromatin state to enable the exploration of the gene expression space ( Antolović *et al.*, 2017; Fritzsch *et al.*, 2018; Nicolas *et al.*, 2017; Zhang & Zhou, 2018).

**Table 1.** Pairwise correlation coefficients between transcriptional uncertainty and transcriptional burst frequency/burst size.

	Correlation with Transcriptional Uncertainty (p-value ≤ 0.01 in red boldface):	
	Burst frequency	Burst size
**Bargaje *et al.***	**0.74**	**0.70**
**Treutlein *et al.***	**0.71**	**0.64**
**Richard *et al.***	**0.68**	0.55
**Stumpf *et al.***	**0.68**	**0.88**
**Moussy *et al.***	0.40	**0.81**
**Guo *et al.***	**0.75**	**0.82**
**Nestorowa *et al.***	**0.73**	**0.71**
**Moignard *et al.***	0.04	**0.83**
**La Manno *et al.***	**0.78**	0.55
**Kreigstein *et al.***	**0.77**	0.32

### RNA velocity and transcriptional uncertainty

In a recent paper ( La Manno *et al.*, 2018), La Manno and colleagues introduced the concept of RNA velocity, which involves computing the rate of change of mRNA from the ratio of unspliced to spliced mRNA. A positive RNA velocity indicates an induction of gene expression, while a negative RNA velocity indicates a repression of gene expression. La Manno *et al.* demonstrated that RNA velocities are able to predict the trajectory of cells undergoing a dynamical transition, such as in circadian rhythms or cell differentiation. In the following, we explored the relationship between RNA velocities and single-cell transcriptional uncertainty.

**Figure 3.**
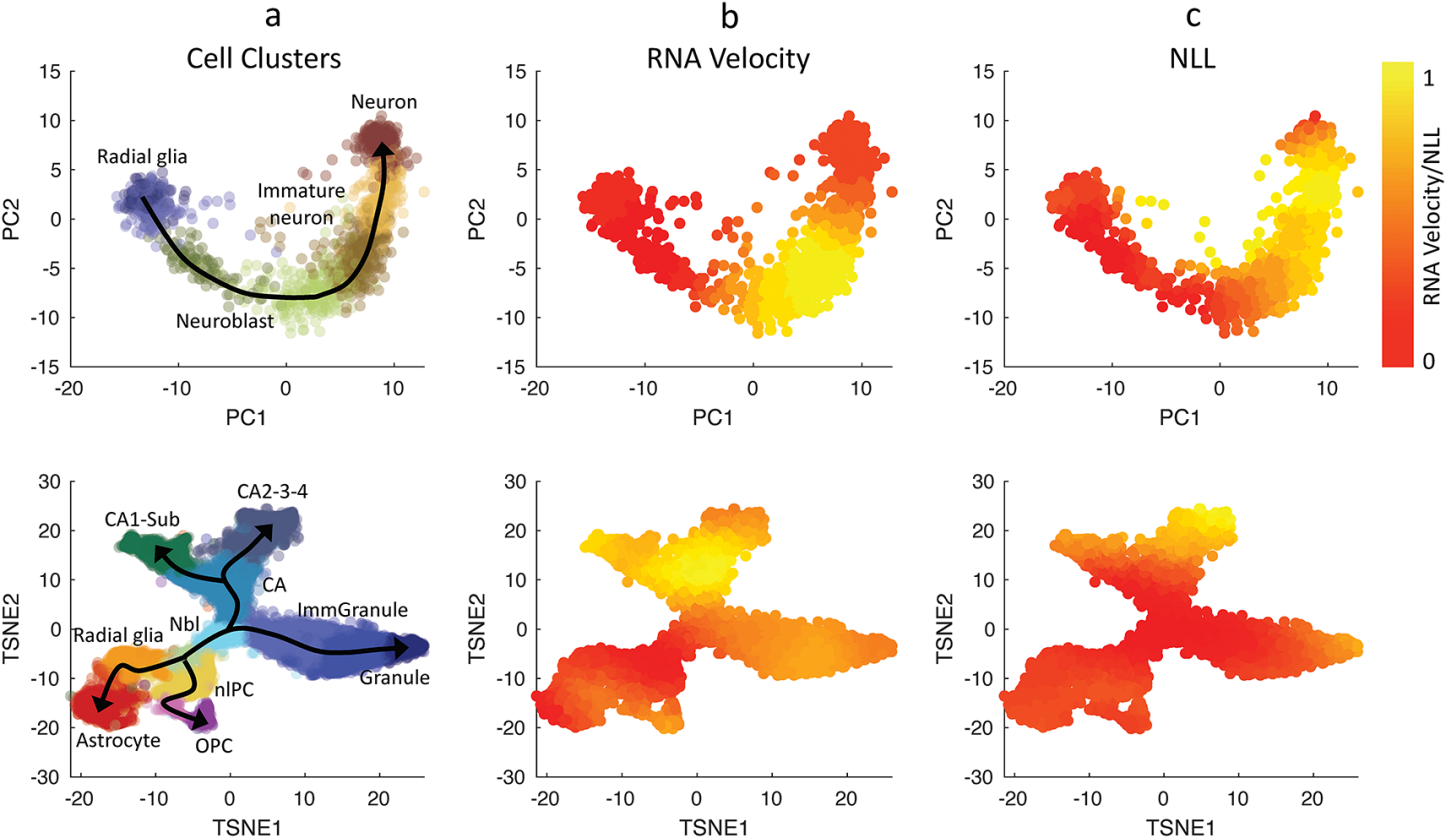
Comparison between RNA velocities estimated using Velocyto and CALISTA negative log-likelihood (NLL) values. (Top row) Human glutamatergic neurogenesis and (Bottom row) mouse hippocampal neurogenesis ( La Manno *et al.*, 2018). (First column) Cell clustering assignments evaluated from Velocyto ( La Manno *et al.*, 2018). Normalized values for Euclidean norm of RNA velocities (2 ^nd^ column), CALISTA single-cell transcriptional uncertainty (NLL; 3 ^rd^ column). The colors in the first column indicate the cell clusters, and those in the second-third columns indicate the normalized cell-wise RNA velocities and NLL values respectively.

We evaluated the single-cell transcriptional uncertainty and RNA velocity for two single-cell gene expression datasets that were previously analyzed in La Manno *et al.* (2018). The first dataset came from human glutamatergic neurogenesis which has a linear (non-bifurcating) lineage progression. Figure 3 (top row) depicts the cell clustering, single-cell transcriptional uncertainty, and RNA velocities (see also *Extended data,* S13 Figure ( Gao *et al.*, 2023)). The single-cell transcriptional uncertainty landscape again has the rise-then-fall shape, as in the other cell differentiation systems discussed above. Interestingly, the same rise-then-fall profile is also seen in the RNA velocity. As illustrated in Figure 3, the increase and decrease of the RNA velocity preceed the transcriptional uncertainty, and the peak of RNA velocity occurs prior to those of the transcriptional uncertainty (see the *Extended data* S1 File for animated illustration ( Gao *et al.*, 2023)). Furthermore, a gene-wise cross-correlation analysis confirms a positive correlation between RNA velocity and single-cell transcriptional uncertainty with a delay for individual genes (see the *Extended data,* Figure S14 ( Gao *et al.,* 2023)).

We also compared RNA velocity and single-cell transcriptional uncertainty for another dataset from mouse hippocampal neurogenesis with a multi-branching lineage ( La Manno *et al.*, 2018). Figure 3 (bottom row) shows that like in the neurogenesis dataset earlier, the RNA velocity increases and then decreases during cell differentiation, and the change in the RNA precede that of the transcriptional uncertainty (see the *Extended data,* S2 File for animated illustration ( Gao *et al.*, 2023)). Also, the RNA velocity peaks take place before the transcriptional uncertainty peaks. The rise-then-fall dynamic of the RNA velocity seen in the two datasets above is consistent with the view that cells engage in exploratory stochastic dynamics as they leave the progenitor state, and disengage this explorative mode as they reach toward the final cell state.

## Discussion

Although Waddington’s epigenetic landscape was originally proposed only as a metaphor, the landscape has helped stem cell researchers to conceptualize the cell differentiation processes through canalization of cell lineages. As mentioned earlier, much of the existing literature on the analytical reconstruction of the epigenetic landscape relied on either a dynamical system theory applied to a simple gene network, or a thermodynamic interpretation based on the potential energy of a reaction ( Bhattacharya *et al.*, 2011; Rebhahn *et al.*, 2014). In this study, we did not make any prior assumptions on the gene regulatory network driving the differentiation process nor on the characteristics of the landscape, such as the existence of a stable valley or that of an energetic barrier (hill). Rather, we assumed that the gene transcription at the single-cell level occurs via stochastic transcriptional bursts that described by a two-state stochastic gene transcription model ( Peccoud & Ycart, 1995). We defined single-cell transcriptional uncertainty based on the likelihood of the cell’s gene expression, computed using the steady-state mRNA distribution from the stochastic transcriptional model above. While high transcriptional uncertainty may reflect a cell with an aberrant gene expression signature with respect to other cells of the same state, such a cell will have little effect on the shape of the transcriptional uncertainy landscape. More importantly, high single-cell transcriptional uncertainty also reflects a cell state that is characterized by high level of heterogeneity in gene transcription. These cells together form the hill region of our transcriptional uncertainty landscape. Thus, the transcriptional uncertainty landscape in our study is a reflection of the dynamic trajectory of gene transcriptional stochasticity during the cell differentiation process.

The two-state model used in CALISTA captures the essential features of stochastic transcriptional bursts – an ON/OFF promoter state and an mRNA transcription only during the ON state. The model is able to reproduce the characteristic negative binomial distribution of mRNA commonly observed in single-cell transcriptomic data. More detailed modelling of gene transcriptional bursts that includes RNA polymerase recruitment and paused release, maturation of nascent mRNA, and cell divisions ( Bartman *et al.*, 2019; Cao *et al.*, 2020; Suter *et al.*, 2011), demonstrates how various aspects of gene transcription contribute to the overt cell-to-cell heterogeneity in gene expression. Under conservative simplifying assumptions, the mRNA distribution from the more detailed models can be reduced to that of the two-state model. Thus, the parameters of the two-state model, for example the rate constants of promoter activation (OFF-to-ON state) and deactivation (ON-to-OFF), should be interpreted as effective constants – i.e. not fundamental biophysical constants – that capture the aggregate impact of various sources of gene transcriptional stochasticity. Note that while we used the two-state model for single-cell clustering and transcriptional uncertainty calculations, as we demonstrated in the iPSC cell differentiation, the rise-then-fall of the transcriptional uncertainty landscape is still valid when using SIMLR clustering algorithm and when using the empirical distribution of mRNA, rather than the two-state model distribution, for computing single-cell transcriptional uncertainty.

The reconstruction of the transcriptional uncertainty landscapes from 10 single-cell transcriptomic datasets of various cell differentiation processes in our study reveals a universal rise-then-fall trajectory in which cells start from a high potency state with a uniform gene expression pattern in the cell population, then progress through transitional cell state(s) marked by increased transcriptional uncertainty (i.e., higher cell-to-cell variability), and eventually reach one of possibly several final cell states with again a uniform gene expression pattern among the cells. Furthermore, the peaks of the transcriptional uncertainty landscape colocalize with forks in the cell lineage. The rise-then-fall in cell uncertainty agrees well with other reports from different cell differentiation systems ( Han *et al.*, 2020; Mojtahedi *et al.*, 2016; Moussy *et al.*, 2017; Richard *et al.*, 2016; Semrau *et al.*, 2017; Stumpf *et al.*, 2017), suggesting that stem cells go through a transition state of high gene expression uncertainty before committing to a particular cell fate. Notably, an increase of variability is a known early warning signal associated with critical transitions in stochastic dynamical systems that are driven by slow, monotonic change in the bifurcation parameter ( Kuehn, 2011; Sarkar *et al.*, 2019). While the results of our analysis are consistent with critical transitions during cell fate commitment in stem cells (see also ( Mojtahedi *et al.*, 2016)), our analysis does not require nor imply this phenomenon. The existence of a hill or barrier during the intermediate stage of cell differentiation has also been proposed in previous studies ( Braun, 2015; Fard *et al.*, 2016; Moris *et al.*, 2016). In particular, Moris and colleagues compared this transition state to the activation energy barrier in chemical reactions ( Moris *et al.*, 2016). We noted however, that a hill in our transcriptional uncertainty landscape is a reflection of a peak in the cell-to-cell gene expression variability, and thus does not represent a resistance or barrier that a cell has to overcome.

In the analysis of iPSCs differentiation into cardiomyocytes ( Bargaje *et al.*, 2017), the genes that contribute significantly to the overall transcriptional uncertainty at or around the peak in the landscape (clustes 2 and 3 in Figure 1e) are known to regulate cardiomyocyte differentiation ( Bargaje *et al.*, 2017) (see the *Extended data* S1 Table for gene lists and S5 Figure and S6 Figure for pathway enrichment analysis of these genes ( Gao *et al.*, 2023)), supporting the idea that dynamic cell-to-cell variability has a functional role in cell-fate decision making processes ( Guillemin *et al.*, 2018; Moris *et al.*, 2018; Rebhahn *et al.*, 2014). Such an idea would be in congruence with the recent demonstration that, in a physiologically relevant cellular system, gene expression variability is functionally linked to differentiation ( Guillemin *et al.*, 2018; Moris *et al.*, 2018).

The rise-then-fall trajectory in the transcriptional uncertainty landscape are more pronounced in some datatsets than in others. For example, in Nestorowa ( Nestorowa *et al.*, 2016) and Moignard ( Moignard *et al.*, 2013) datasets (see Figure 2c), peaks in the transcriptional uncertainty landscape are less noticeable than in the other differentiation systems. We noted that cells in the Nestorowa ( Nestorowa *et al.*, 2016) and Moignard ( Moignard *et al.*, 2013) studies were pre-sorted by using flow cytometry based on the expression of surface protein markers. We posited that at least some cells in the transition state(s) might have been lost during the cell pre-sorting since such cells might not express the chosen surface markers strongly.

Further, the correlation analysis between the cell transcriptional uncertainty and biologically meaningful rates of the stochastic gene transcription model showed strong positive correlations with transcriptional burst size and frequency. Note that cellular processes such as cell division can affect the heterogeneity of mRNA in a cell population in a similar fashion as stochastic gene transcriptional bursts ( Cao & Grima, 2020; Perez-Carrasco *et al.*, 2020), providing an alternate explanation for gene expression fluctuations. But, several studies have reported an increase in gene transcriptional bursts during transition states in cell differentiation and other recent studies have suggested that both burst frequency and burst size regulate gene expression levels ( Antolović *et al.*, 2017; Fritzsch *et al.*, 2018; Zhang & Zhou, 2018). Importantly, our comparison of the single-cell transcriptional uncertainty and the single-cell RNA velocity revealed that an increase (decrease) in RNA velocity predicts an increase (decrease) in transcriptional uncertainty after a short delay, and that a peak of RNA velocity preceeds that of the transcriptional uncertainty.

The aforementioned observations, while correlative in nature, points to possible biological mechanisms underlying the universal dynamic feature of single-cell transcriptional uncertainty during cell differentiation. At the start of the differentiation process, cells engage an exploratory search dynamics in the gene expression space by increasing stochastic transcriptional burst size and burst frequency. The putative objective of such a stochastic search is to optimize the cell’s gene expression pattern given its new environment. The engagement of this stochastic exploratory mode is supported by the observed increased in the overall RNA velocity and its expected-but-delayed effect in elevating the cell-to-cell gene expression variability (i.e. higher transcriptional uncertainty). Increased transcriptional burst size and frequency are an indication of increased frequency of the promoter turning ON (higher *θ _on_* and lower *θ _off_*).

A possible mechanism behind this exploratory search dynamic is an increase in chromatin mobility, driven by metabolic alterations in early differentiation ( Paldi, 2012). Multiple studies have demonstrated that a mismatch between the intracellular state of stem cells and their immediate environment can lead to metabolic reorganization ( Argüello-Miranda *et al.*, 2018; Folmes *et al.*, 2011; Gu *et al.*, 2016). More specifically, a change in the balance between glycolysis and OXPHOS metabolism has been associated to numerous differentiation processes (see ( Richard *et al.*, 2019) and references therein). Furthermore, changes in the metabolic flux state in early differentiation can modulate the activity of chromatin modifying enzymes through their metabolic co-factors ( Moussaieff *et al.*, 2015), or in more direct fashion ( Zhang *et al.*, 2019) and alter the cell differentiation outcome. A more dynamic state of the chromatin is associated with more variable gene expressions due to the changes in the opening-closing dynamics (breathing) of the chromatin ( Zwaka, 2006). As the cells approach the final state, cells disengage the exploratory search mode, as the cells approach an optimal gene expression and metabolic state associated with a chosen cell type.

The findings of our analysis fit within the paradigm of a stochastic stem cell differentiation process. More specifically, in this paradigm, the cell differentiation is thought to proceed as follows ( Braun, 2015; Kupiec, 1996, 1997; Paldi, 2003):

I) extrinsic and/intrinsic internal stimuli, such as a medium change or the addition of new molecules in the external medium, trigger a cellular response that destabilizes the initial high potency cell state;
II) each cell alters its internal cell state and engages an exploratory dynamic through a combination of the inherent stochastic dynamics of gene transcription and the emergence of new stable cell state(s). At the cell population level, we observe a rise in the cell-to-cell variability of gene expression;
III) a physiological selection/commitment to one stable lineage among possibly multiple lineages;
IV) finally, a reduction in the exploratory dynamics commences along with the establishment of stable cell state(s) corresponding to differentiated cell type(s).

The disordered gene expression pattern during the transition period can be seen as an exploratory dynamic to find the optimal pattern(s) ( Braun, 2015; Paldi, 2003). The transcriptional uncertainty in our analysis can be interpreted as the width of the valley in Waddington’s epigenetic landscape. If one considers the epigenetic landscape as a depiction of the accessible gene expression subspace through which stochastic single-cell trajectories pass during differentiation, a wider valley indicates a more variable gene expression pattern. While in the original Waddington’s epigenetic landscape the valley naturally widens around the branching point in the cell lineage, our analysis shows that a widening of the valley (an increase in transcriptional uncertainty) also occurs in non-branching lineage. In other words, the increase in transcriptional uncertainty appears to be a universal feature of the cell differentiation process, one that arises from the engagement of exploratory mode through increased stochasticity in transcriptional bursts, as explained above. The above view is also compatible with the idea that cell phenotype transition results from the dynamics of an underlying stochastic molecular network ( Gupta *et al.*, 2011; Thomas *et al.*, 2014).

In summary, our model-based single-cell transcriptome analysis and the evaluation of single-cell transcriptional uncertainty have shed a new light on the role of stochastic dynamics of gene transcription in the cell differentiation process. Importantly, the peaks of single-cell transcriptional uncertainty mark cellular decision-making points in the cell lineage tree. By identifying, isolating, and analyzing more comprehensively individual cells from the peaks of transcriptional uncertainty, we can gain a much better understanding of the key molecular players in the stem cell decision-making.

## Data Availability

All the public single cell data sets analysed in this study are available from the original publications ( Bargaje *et al.*, 2017; Guo *et al.*, 2010; La Manno *et al.*, 2018; Moignard *et al.*, 2013; Moussy *et al.*, 2017; Nestorowa *et al.*, 2016; Richard *et al.*, 2016; Stumpf *et al.*, 2017; Treutlein *et al.*, 2016). Zenodo: Extended Data for Single-cell Transcriptional Uncertainty Landscape of Cell Differentiation. https://doi.org/10.5281/zenodo.7776102 ( Gao *et al.*, 2023). This project contains the following underlying data:
• Data file 1. Additional Figures and Notes (S1-S14 Fig. and S1 Note of CALISTA workflow) • Data file 2. S1 Table. Genes with high transcriptional uncertainty in Cluster 2 and 3 of Bargaje et al. ( Bargaje *et al.*, 2017) data analysis. • Data file 3. S1 File. Animated illustration of RNA velocity and transcriptional uncertainty landscape in mouse hippocampal neurogenesis ( La Manno *et al.*, 2018). • Data file 4. S2 File. Animated illustration of RNA velocity and transcriptional uncertainty landscape in human glutamatergic neurogenesis ( La Manno *et al.*, 2018). Data are available under the terms of the Creative Commons Attribution 4.0 International license (CC-BY 4.0).

## Software availability

CALISTA tutorial is available from: https://www.cabselab.com/calista

CALISTA source code is available from: https://github.com/CABSEL/CALISTA

License: BSD-3-Clause license
