# Supplementary material for: Single-cell transcriptional uncertainty landscape of cell differentiation
Source: F1000Res. 2023 Jul 20;12:426. Originally published 2023 Apr 20. [Version 2] doi: 10.12688/f1000research.131861.2 (PMC10400935; doi:10.12688/f1000research.131861.2)
Supplement: Peer review history

# Peer review: Reviewer response for version 2

**DOI:** 10.5256/f1000research.153194.r188744

**Published:** 2023-08-03

**Authors:** Geneviève Dupont

**Affiliations:** 1 Unité de Chronobiologie Théorique, Faculté Des Sciences, Universite Libre de Bruxelles, Brussels, Brussels, Belgium

I thank the authors for their changes. I do not have any additional comments.

Is the work clearly and accurately presented and does it cite the current literature?

Yes

If applicable, is the statistical analysis and its interpretation appropriate?

I cannot comment. A qualified statistician is required.

Are all the source data underlying the results available to ensure full reproducibility?

Yes

Is the study design appropriate and is the work technically sound?

Yes

Are the conclusions drawn adequately supported by the results?

Yes

Are sufficient details of methods and analysis provided to allow replication by others?

Partly

Reviewer Expertise:

Modelling differentiation processes

I confirm that I have read this submission and believe that I have an appropriate level of expertise to confirm that it is of an acceptable scientific standard.

# Peer review: Reviewer response for version 1

**DOI:** 10.5256/f1000research.144746.r170464

**Published:** 2023-05-09

**Authors:** Geneviève Dupont

**Affiliations:** 1 Unité de Chronobiologie Théorique, Faculté Des Sciences, Universite Libre de Bruxelles, Brussels, Brussels, Belgium

Using the CALISTA method that they have developed, Papili Gao *et al*. quantify single cell transcriptional uncertainty in 10 sets of single cell data associated to differentiating systems. This includes single-cell mRNA as well as single-cell RT-qPCR. The representation of the cells in the uncertainty landscape allows to visualize the differentiation trajectories and the increase in stochasticity that precedes each fate commitment.  The authors also show that the transcriptional uncertainty correlates with the RNA velocity, a concept that was introduced by La Manno *et al*. in 2018.

 These results shed light on the mechanism underlying cell fate commitment, with the increase of uncertainty allowing cells to possibly engage in different cellular outcomes. It also provides a new data-based visualisation of the Waddington's landscape that directly takes stochasticity in gene expression into account.

 I would suggest the authors to clarify how the cell likelihood values are computed from the data, because it is central to the work and its conclusions. In my opinion, the section entitled “Pairwise correlation analysis …” should be extended. In particular, the link with the parameters of the 2 state models should be made explicit. In the same line, the fit of the data with the distributions of the 2 state model may be discussed.

 The authors also may wish to comment about the possible biological significance of the depths of the different valleys, as observed for example in the linear trajectories of the first line of Figure 2C (early embryonic development in mice).

 In the Introduction, the work by M. Saez and colleagues ^1^ should be added in the paragraph related to the graphical representation of the Waddington's landscape.

Is the work clearly and accurately presented and does it cite the current literature?

Yes

If applicable, is the statistical analysis and its interpretation appropriate?

I cannot comment. A qualified statistician is required.

Are all the source data underlying the results available to ensure full reproducibility?

Yes

Is the study design appropriate and is the work technically sound?

Yes

Are the conclusions drawn adequately supported by the results?

Yes

Are sufficient details of methods and analysis provided to allow replication by others?

Partly

Reviewer Expertise:

Modelling differentiation processes

I confirm that I have read this submission and believe that I have an appropriate level of expertise to confirm that it is of an acceptable scientific standard, however I have significant reservations, as outlined above.

## References

[1] : Statistically derived geometrical landscapes capture principles of decision-making dynamics during cell fate transitions. *Cell Syst* .2022;13(1) : 10.1016/j.cels.2021.08.013 12-28.e3 10.1016/j.cels.2021.08.013 34536382PMC8785827

# Response

**Published:** 2023-07-18

**Authors:** Rudiyanto Gunawan

**Affiliations:** Chemical and Biological Eng., University at Buffalo, Buffalo, New York, USA

**Comment:**

 Using the CALISTA method that they have developed, Papili Gao *et al*. quantify single cell transcriptional uncertainty in 10 sets of single cell data associated to differentiating systems. This includes single-cell mRNA as well as single-cell RT-qPCR. The representation of the cells in the uncertainty landscape allows to visualize the differentiation trajectories and the increase in stochasticity that precedes each fate commitment.  The authors also show that the transcriptional uncertainty correlates with the RNA velocity, a concept that was introduced by La Manno *et al*. in 2018.

 These results shed light on the mechanism underlying cell fate commitment, with the increase of uncertainty allowing cells to possibly engage in different cellular outcomes. It also provides a new data-based visualisation of the Waddington's landscape that directly takes stochasticity in gene expression into account.

**Response:**

 On behalf of my co-authors, I would like to sincerely thank the reviewer for the critiques on our work. We revised the manuscript to address the reviewer’s comments. Below, I am providing the response to each of the comments.

**Comment:**

 I would suggest the authors to clarify how the cell likelihood values are computed from the data, because it is central to the work and its conclusions. In my opinion, the section entitled “Pairwise correlation analysis …” should be extended. In particular, the link with the parameters of the 2 state models should be made explicit. In the same line, the fit of the data with the distributions of the 2-state model may be discussed.

**Response:**

 Following the suggestion of the reviewer, the section on pairwise correlation analysis has been expanded. In addition, more detail of the computation of the negative log-likelihood for each cell—a metric for single-cell transcriptional uncertainty—is provided in the section “Single-cell transcriptional uncertainty”. The full description of the cell likelihood calculation can be found in the journal article of the method CALISTA ( https://doi.org/10.3389/fbioe.2020.00018).

 Regarding the fit of the data to the theoretical distribution from the 2-state model, we noted that the transcriptional uncertainty was computed for each cell and therefore it was not possible to evaluate distribution fit to the 2-state model. Still, we may compare the gene expression distribution of cells that were initially assigned to clusters. An example of the distribution fit using Kolmogorov-Smirnov (KS) distance—the maximum difference in the cumulative distributions between the data and theoretical distribution—is shown in Figure A.

 Figure A. Kolmogorov-Smirnov distances of single cell gene expression distribution. The distances were computed between the distribution of gene expression from CALISTA clusters and the theoretical distribution from the 2-state model.

**Comment:**

 The authors also may wish to comment about the possible biological significance of the depths of the different valleys, as observed for example in the linear trajectories of the first line of Figure 2C (early embryonic development in mice).

**Response:**

 Thank you for this comment. We avoided making deeper interpretation and comparison of the depth of the valley—and by the same token, the height of the hill—across datasets because the negative log-likelihood values were not normalized. One reason was that different datasets include distinct sets of genes. In addition, the experimental platforms that were used to generate single-cell gene expression data differ across datasets. One potential avenue to produce comparable landscapes across datasets would be to use a common mechanistic model describing gene transcriptional network as we recently described in ( https://doi.org/10.1371/journal.pcbi.1010962).

**Comment:**

 In the Introduction, the work by M. Saez and colleagues should be added in the paragraph related to the graphical representation of the Waddington's landscape.

**Response:**

 Following the reviewer’s suggestion, we have added this reference in Introduction.

# Peer review: Reviewer response for version 1

**DOI:** 10.5256/f1000research.144746.r170465

**Published:** 2023-04-24

**Authors:** Alessandro Giuliani

**Affiliations:** 1 Environment and Health Department, Istituto Superiore di Sanità, Rome, Italy

In this paper the authors are able to 'put on the ground' the time-honoured Waddington's metaphor of epigenetic landscape. They were not the first ones to face the problem to make quantitative and operational the Waddington model but, at odds with other scholars, Gao *et al.* offer the readers a very clear and immediately reproducible data analytic procedure free of any strong theoretical frame, being only based on classical multivariate data analysis structural optimization principles with no use of a priori distributional hypothesis. Thus, the 'amount of genome expression uncertainty' at both single cell and population levels only descends from the degradation of structural compactness of clusters in terms of within cluster distance following cluster fusion and by the decrease in cell-cell correlation.

 In this way the authors are able to uncover the 'B side' of Waddington landscape, i.e., the 'ropes' underlying the landscape and provoking its changes in shape (see Gigante, Giuliani, and Mattia 2023 ^1^).

 By the analysis of diverse data sets, the authors are able to answer their initial question about the 'bystander' or 'essential' character of the observation of an 'entropic burst' for initiating the trajectory going from a relatively stable initial stemness attractor toward cell terminal differentiation state, by a clear proof of the unescapable need of such entropic burst.

 The material basis of such entropic burst must be looked for in the chromatin decompaction allowing for an increased variance of gene expression that was already observed by other scholars (see for example Zimatore *et al.,* 2021 ^2^).

 All in all, this is a very elegant and innovative work that opens new avenues to the analysis of cell differentiation process by means of a physically plausible model.

Is the work clearly and accurately presented and does it cite the current literature?

Yes

If applicable, is the statistical analysis and its interpretation appropriate?

Yes

Are all the source data underlying the results available to ensure full reproducibility?

Yes

Is the study design appropriate and is the work technically sound?

Yes

Are the conclusions drawn adequately supported by the results?

Yes

Are sufficient details of methods and analysis provided to allow replication by others?

Yes

Reviewer Expertise:

NA

I confirm that I have read this submission and believe that I have an appropriate level of expertise to confirm that it is of an acceptable scientific standard.

## References

[1] : A novel network approach to multiscale biological regulation. *Cell Syst* .2023;14(3) : 10.1016/j.cels.2023.02.004 177-179 10.1016/j.cels.2023.02.004 36924765

[2] : Self-organization of whole-gene expression through coordinated chromatin structural transition. *Biophysics Reviews* .2021;2(3) : 10.1063/5.0058511 10.1063/5.0058511 PMC1090350438505632

# Response

**Published:** 2023-05-02

**Authors:** Rudiyanto Gunawan

**Affiliations:** Chemical and Biological Eng., University at Buffalo, Buffalo, New York, USA

On behalf of the co-authors, I wish to thank the reviewer for the positive feedback and comments on our work.
